# Post-traumatic stress disorder: a need for alternative approaches

**DOI:** 10.1192/j.eurpsy.2025.1948

**Published:** 2025-08-26

**Authors:** M. V. Chiappe

**Affiliations:** 1 University of Medicine. Pharmacological Chair UBA; 2 Hospital de Emergencias Psiquiatricas Alvear, Buenos Aires, Argentina

## Abstract

**Introduction:**

Post-traumatic stress disorder (PTSD) is a mental health statue that arises from traumatic events featuring by intrusive memories of the incident, difficulty in interpersonal relationships, recurrent distress, avoidance of similar situations and flashbacks. Although conventional treatments such as cognitive behavioral therapy (CBT), exposure therapy, selective serotonin reuptake inhibitors (SSRIs), and medications like prazosin are widely used and supported by scientific evidence, an increasing number of individuals are turning to alternative treatments. For instance, mind-body practices have demonstrated therapeutic benefits for stress reduction, including decreased anxiety, depression, and anger, as well as improved pain tolerance, self-esteem, relaxation, and coping abilities. Practices such as yoga, tai chi, and meditation, which involve physical postures, stretching, and deep breathing, are gaining popularity for managing PTSD symptoms. Additionally, lifestyle changes, diet, mindful breathing, Ayurvedic medicine, and somatic therapies like dance therapy are increasingly recognized as valuable tools for improving quality of life in trauma survivors Clinicians should discuss mind-body interventions with their patients and educate them about the potential benefits of them to maximize the diversity of treatment options.

**Objectives:**

This review aims to explore both conventional and alternative approaches for PTSD treatment, including psychotherapy, psychopharmacology, and complementary practices such as exercise, diet, meditation, yoga, deep breathing, and dance therapy.

**Methods:**

The study is a literature review conducted through PubMed.

**Results:**

The studies included in this review exhibited considerable heterogeneity, with varying designs, intervention methods, and study durations, as well as differing use of control groups. As a result, conducting a true meta-analysis was not feasible.

**Conclusions:**

“Mind-body practices” incorporate numerous therapeutic effects on stress responses, including reductions in anxiety, depression, and anger, and increases in pain-tolerance, self-esteem, energy levels, ability to relax, and ability to cope with stressful situations.

Given the limitations in the current evidence for conventional treatments and the growing use of complementary and alternative approaches, there is a pressing need for more comprehensive re-search into these alternative therapies. Future studies should aim to evaluate the effectiveness of mind-body interventions and other complementary practices as potential treatments for PTSD.

**Disclosure of Interest:**

None Declared

